# Neurons Controlling *Aplysia* Feeding Inhibit Themselves by Continuous NO Production

**DOI:** 10.1371/journal.pone.0017779

**Published:** 2011-03-09

**Authors:** Nimrod Miller, Ravit Saada, Shlomi Fishman, Itay Hurwitz, Abraham J. Susswein

**Affiliations:** The Leslie and Susan Gonda (Goldschmied) Multidisciplinary Brain Research Center, and The Mina and Everard Goodman Faculty of Life Sciences, Bar Ilan University, Ramat Gan, Israel; Freie Universitaet Berlin, Germany

## Abstract

**Background:**

Neural activity can be affected by nitric oxide (NO) produced by spiking neurons. Can neural activity also be affected by NO produced in neurons in the absence of spiking?

**Methodology/Principal Findings:**

Applying an NO scavenger to quiescent *Aplysia* buccal ganglia initiated fictive feeding, indicating that NO production at rest inhibits feeding. The inhibition is in part via effects on neurons B31/B32, neurons initiating food consumption. Applying NO scavengers or nitric oxide synthase (NOS) blockers to B31/B32 neurons cultured in isolation caused inactive neurons to depolarize and fire, indicating that B31/B32 produce NO tonically without action potentials, and tonic NO production contributes to the B31/B32 resting potentials. Guanylyl cyclase blockers also caused depolarization and firing, indicating that the cGMP second messenger cascade, presumably activated by the tonic presence of NO, contributes to the B31/B32 resting potential. Blocking NO while voltage-clamping revealed an inward leak current, indicating that NO prevents this current from depolarizing the neuron. Blocking nitrergic transmission had no effect on a number of other cultured, isolated neurons. However, treatment with NO blockers did excite cerebral ganglion neuron C-PR, a command-like neuron initiating food-finding behavior, both in situ, and when the neuron was cultured in isolation, indicating that this neuron also inhibits itself by producing NO at rest.

**Conclusion/Significance:**

Self-inhibitory, tonic NO production is a novel mechanism for the modulation of neural activity. Localization of this mechanism to critical neurons in different ganglia controlling different aspects of a behavior provides a mechanism by which a humeral signal affecting background NO production, such as the NO precursor L-arginine, could control multiple aspects of the behavior.

## Introduction

Release of the unconventional neurotransmitter nitric oxide (NO) is contingent on the activity of an enzyme, nitric oxide synthase (NOS), rather than on depolarization-dependent vesicle release. NO is generally released because NOS is activated by Ca^2+^ entry into the cell when it spikes [1]. However, if NOS were active even without spikes, NO release via the actions of NOS could modulate neurons without neural activity. We have examined the possible control of key neurons affecting *Aplysia* feeding by NO in the absence of spiking.

*Aplysia* feeding is a complex behavior that consists of appetitive (food finding) behaviors controlled primarily by the cerebral ganglion, and subsequent consummatory behaviors controlled primarily by the buccal ganglia [2], [3]. NO is an established transmitter in both the cerebral and buccal ganglia of *Aplysia* and other gastropod molluscs [4]–[15], and in these ganglia NO has been shown to be released from nitrergic neurons when they fire.

In a number of other systems, in addition to being released in response to a stimulus, NO also acts as a tonic modulator of neural activity, and its tonic modulation is revealed when the actions of NO are blocked [11], [16]–[20]. We examined the possibility that in addition to being released by stimuli signaling aspects of feeding, NO is also produced in the absence of elicited neural activity in the *Aplysia* buccal ganglia. We found that inhibition of NO actions in the buccal ganglia initiates fictive feeding in the absence of additional stimuli. Thus, as in other systems, NO is a tonic modulator of the central pattern generator (CPG) generating repetitive feeding behaviors.

Access to major elements of the CPG organizing *Aplysia* consummatory feeding behaviors [21] allowed us to investigate the loci at which tonic NO production regulates feeding. In particular, we were able to examine possible effects of NO on B31/B32, key neurons having a central role in deciding to initiate consummatory feeding behaviors [22], [23]. We found that B31/B32 is inhibited by NO. The ability to study *Aplysia* CPG neurons cultured in isolation [24] allowed us to examine some of the cellular mechanisms by which NO acts on B31/B32. Such experiments showed that these neurons produce NO at rest, and NO contributes to their resting potential. Blockers of NO opened a depolarizing leak current, suggesting that NO acts at rest to block this current.

Regulating neural activity via the background production of a neuroactive agent such as NO could potentially act as a mechanism to coordinate different aspects of a behavior that are controlled at different neural sites. For example, NO could be produced in the absence of neural activity by neurons controlling food finding in the cerebral ganglion and food consumption in the buccal ganglia. A circulating metabolite or a hormone could then affect both these sites, and thereby regulate multiple aspects of feeding behavior. This and an additional paper [25] demonstrate such regulation. In this paper, we show that nitrergic self-inhibition is found in buccal ganglia neurons B31/B32, which control consummatory behaviors [26], [27], [28], as well as in neuron C-PR, a command neuron for a behavioral state, food arousal [29], [30].

## Results

### Background NO production in the buccal ganglia inhibits feeding programs

**Figure 1:**
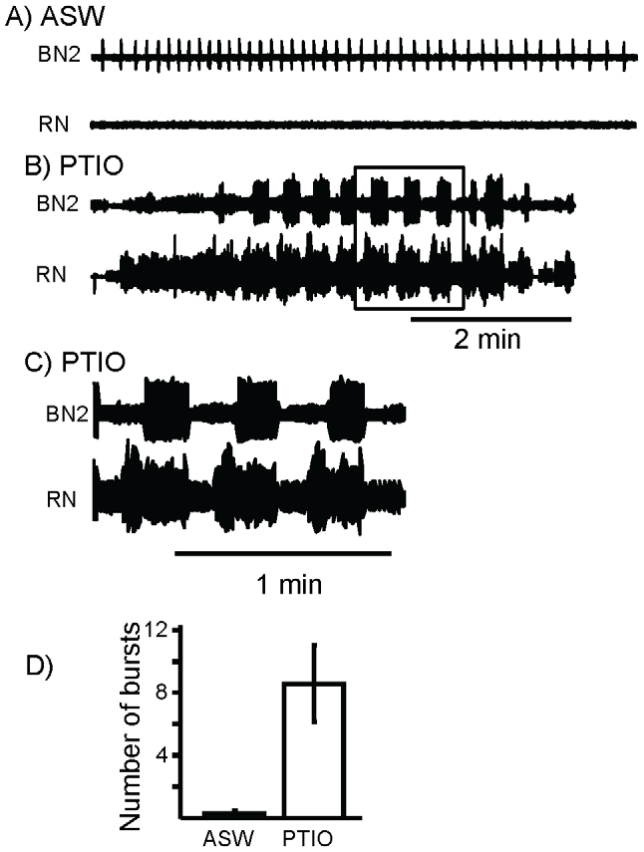
The NO scavenger PTIO induces fictive feeding when applied to the isolated buccal ganglia. Fictive feeding was monitored via extracellular recordings from the radula nerve (RN) and from buccal nerve 2 (BN2). Activity in RN is a correlate of radula closing, whereas activity in BN2 is a correlate of retraction. Activity representative of radula retraction was counted as a single burst of fictive feeding. **A**) In ASW, no fictive feeding was seen, although a single unit in BN2 fired. **B**) Application of PTIO (at the start of the trace) elicited repeated bursts of fictive feeding. Recordings similar to those shown were observed in 7 separate isolated buccal ganglia preparations. **C**) Expansion of the boxed area in part B shows overlap between firing in BN2 and RN, indicating that PTIO induced ingestion-like activity. **D**) Means and standard errors of the number of fictive feeding bursts recorded in 10 min in ASW and after the application of PTIO. PTIO caused a significant increase in fictive feeding (*p* = 0.02 *t*(6) = 2.78; two-tailed paired *t*-test).

Isolated buccal ganglia in *Aplysia* contain a central pattern generator (CPG) organizing consummatory feeding behaviors. Activation of the CPG causes fictive feeding consisting of protraction and retraction phases of activity [21]. Fictive feeding can be monitored via extracellular recordings from buccal nerves [31], as well as via intracellular recordings from neurons B31/B32 [26], [27]. Since NO is a modulator of central pattern generators in other systems [11], [17], [18], [20], [32], and since NO affects aspects of *Aplysia* feeding [8], we examined the possibility that NO production within the buccal ganglia has a role in modulating the buccal ganglia CPG. Treating the isolated ganglia with the NO scavenger PTIO induced fictive feeding consisting of repetitive cycles of protraction and retraction, as monitored via extracellular recordings from buccal nerves (Fig. 1). In the extracellular recording shown, there were no bursts of fictive feeding in ASW (Fig. 1A). However, application of PTIO elicited fictive feeding (Fig. 1B, D). The buccal ganglia can produce ingestion-like or egestion-like activity [31], [33], [34]. In ingestion-like activity, the radula closes during retraction, pulling food into the mouth [31]. Firing in the Radula Nerve (RN) is a monitor of radula closing, whereas firing in Buccal Nerve 2 (BN2) is a monitor of radula retraction [31]. Motor programs elicited by PTIO were ingestion-like (Fig. 1C), as shown by the simultaneity of RN and BN2 activity.

**Figure 2:**
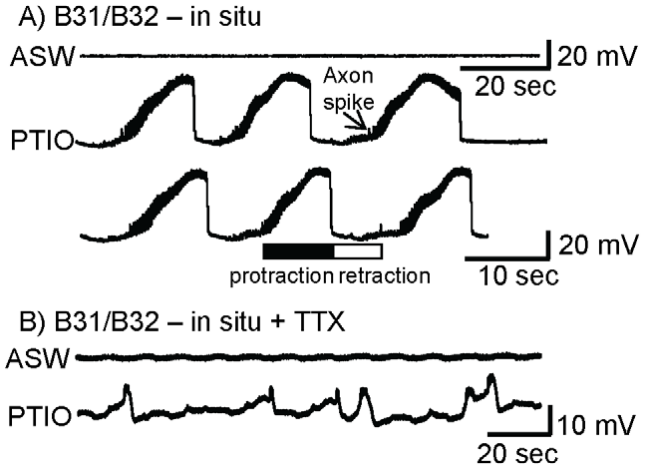
Blocking NO activity elicits fictive feeding as monitored via intracellular recording from neurons B31/B32. **A**) IN ASW, there were no bouts of fictive feeding. Application of the NO scavenger PTIO in 4 of 4 preparations caused fictive consummatory responses, as evidenced by cyclic depolarizations and repolarizations in a B31/B32 neuron. The recordings are a continuous record displaying 6 bouts of fictive feeding over a period of approximately 100 sec. The protraction and retraction phases of fictive feeding are marked. B31/B32 fires during protraction, and is repolarized in retraction. The B31/B32 soma is inexcitable, and axon spikes are recorded in the soma as 5–10 mV spikes, as shown. **B**) PTIO was applied to buccal ganglia in which spiking was blocked by TTX. PTIO elicited cyclical depolarizations of B31/B32 in 4 B31/B32 neurons from 2 preparations. The fast potentials at the top of a depolarization are presumably electrical EPSPs derived from Ca^+2^ spikes in the terminal of the electrically coupled B63 neuron, which fires in response to the depolarization of B31/B32.

Fictive feeding can also be monitored via intracellular recording from key CPG neurons B31/B32 [27]. These neurons depolarize preceding the protraction phase, and remain depolarized throughout protraction. They are repolarized during retraction. The somata of B31/B32 are electrically inexcitable [35], and 10 mV axon spikes are recorded in the soma while B31/B32 is depolarized [26]. B31/B32 is excited by neuron B63 via fast and slow cholinergic synapses, as well as via an electrical synapse [24], [36]. The electrical coupling between B63 and B31/B32 allows B63 to excite B31/B32 as a result of B31/B32 depolarization, which elicits spikes in B63. Application of PTIO to the buccal ganglia caused cyclical depolarizations and repolarizations in B31/B32 (Fig. 2A) typical of that seen previously in response to food, or in response to activation of command-like neurons responding to food [27], [37].

Because no stimuli other than the NO scavenger were used to elicit ficitive feeding, these experiments indicate that NO within the buccal ganglia in the absence of stimuli that elicit feeding is an inhibitory modulator of the buccal ganglia CPG. Reducing the background NO levels by the NO scavenger presumably initiated cyclical B31/B32 activity and fictive feeding by eliminating the inhibitory modulation. Feeding activity in the buccal ganglia elicited by food or other stimuli presumably elicit feeding against a background presence of NO that inhibits feeding.

### NO is produced without spiking by neurons B31/B32

**Figure 3:**
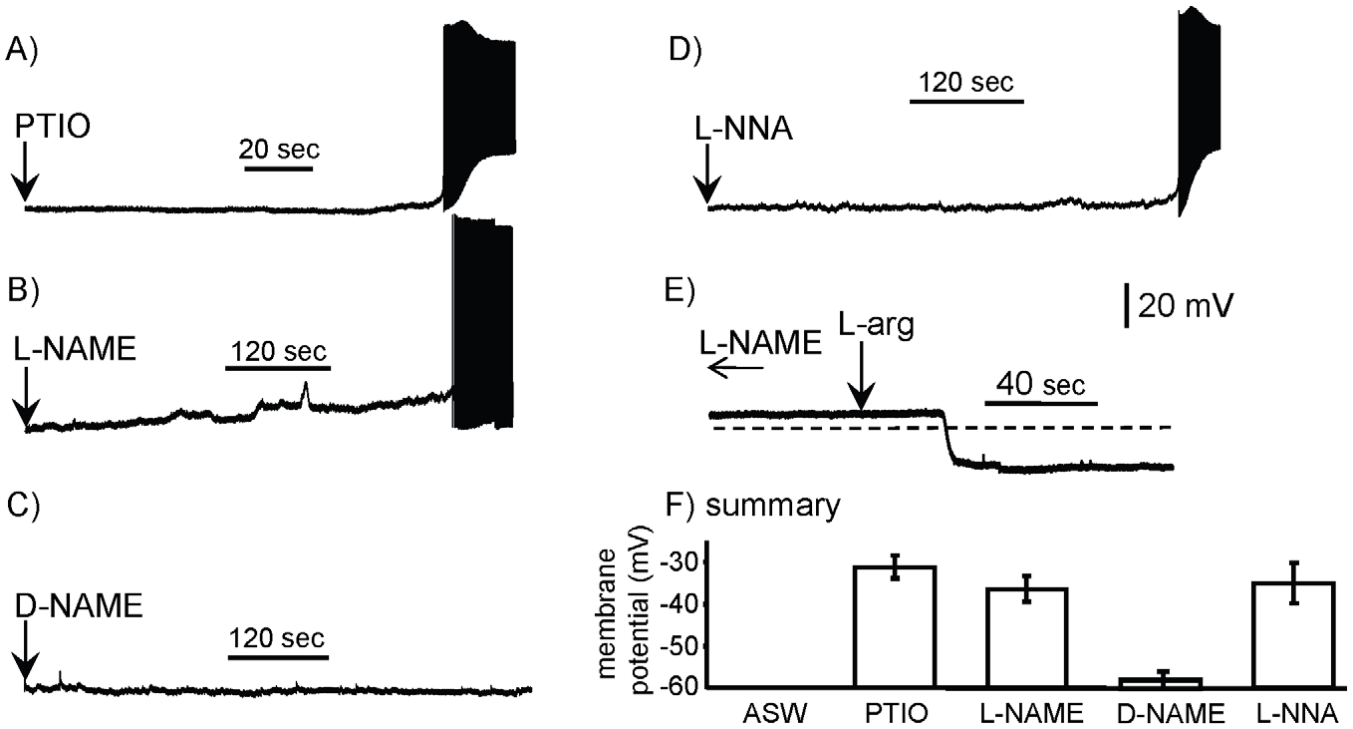
Nitrergic self-inhibition in isolated B31/B32 neurons. In the recordings shown in A–D, pharmacological agents were applied at the arrow, with the resting potential preceding treatment at −60 mV. Note that recordings are minutes in length, reflecting the relatively slow effects caused by the pharmacological agents. **A**) The NO scavenger PTIO depolarized the cell in 5 of 5 preparations. Note that spikes in cultured B31/B32 neurons are larger than in neurons *in situ*. **B**) The NOS inhibitor L-NAME depolarized B31/B32 in 11 of 11 preparations. **C**) In 4 of 5 preparations, D-NAME, the inactive enantiomer of L-NAME, did not depolarize isolated B31/B32 neurons. **D**) A second NOS blocker, L-NNA, depolarized B31/B32 in 4 of 4 preparations. **E**) L-arginine has effects that are opposite to those of blocking NO. In 5 of 5 preparations previously treated with L-NAME, L-arginine (×100 of the L-NAME concentration) reversed the effect of L-NAME. In the example shown the previous treatment with L-NAME (depicted by the arrows to the left) caused a modest depolarization from −60 mV. L-arginine was applied before the cell depolarized fully. The dashed line shows −60 mV. **F**) Summary of the effects of ASW (N = 3), and of D-NAME and of 3 blockers of nitrergic transmission on the membrane potential of B31/B32. For substances causing firing of B31/B32, the potential just before the first spike is shown. Means and standard errors are shown.

B31/B32 are key components of the CPG organizing feeding [26], [35]. Are B31/B32, which are cyclically activated by an NO scavenger, directly affected by the scavenger, or is the effect of the scavenger via activation of neurons that synapse onto B31/B32? To answer this question, we applied PTIO to buccal ganglia that had been treated with tetrodotoxin (TTX), thereby blocking action potentials and synaptic release dependent on Na^+^-dependent spiking (Fig. 2B). If PTIO directly acts on B31/B32, it should depolarize the cell even in the presence of TTX. In the presence of TTX, PTIO elicited cyclical depolarizations of B31/B32 which were followed by repolarizations. The depolarizations are presumably the result of PTIO, indicating that the effect of PTIO is not dependent on spiking and transmitter release. However, PTIO did not depolarize B31/B32 as strongly as it did in the absence of TTX, indicating that TTX partially blocked the effect of PTIO. Block by TTX of the effects of PTIO need not arise as a result of an effect of NO on sodium or potassium currents underlying spiking. The lack of complete depolarization of B31/B32 is also explained by previous findings [22], [24] that the sustained depolarization of B31/B32 is not dependent on an endogenous current, but rather it is driven by synaptic output, both from neurons electrically coupled to B31/B32 and from B31/B32 onto itself. The self-excitatory depolarization of B31/B32 is blocked by TTX. In the absence of TTX, when B31/B32 is sufficiently depolarized, it begins spiking, and excites itself. In the presence of TTX the self-excitatory transmitter release is absent, and endogenous potassium currents [28] can repolarize B31/B32. The cyclical B31/B32 activity is likely to be explained by a direct depolarizing effect of PTIO on B31/B32, combined with repolarizing effects of endogenous K^+^ currents [28]. The PTIO presumably depolarized B31/B32, and thereby activated the K^+^ currents, which repolarized B31/B32. The cycle repeats when the K^+^ currents are deactivated by the repolarization. In the absence of TTX, these potassium currents slow the depolarization, and partially brake it [28], but do not block the transmitter-dependent depolarization. This experiment alone does not eliminate the possibility NO affects cells presynaptic to B31/B32, or that Ca^+2^ dependent spikes which are not blocked by TTX are releasing NO at rest from neurons presynaptic to B31/B32. To be certain that the effects of PTIO on B31/B32 are direct, we examined the effect of PTIO on isolated, cultured B31/B32 neurons (Fig. 3). In 5 of 5 preparations, PTIO depolarized and caused firing in isolated B31/B32 neurons (Fig. 3A). Since no other neuron was present, and no spikes were observed in the absence of stimuli, PTIO must have a direct effect on B31/B32. Since it depolarized the cell in the absence of spiking, this result cannot be explained by Ca^+2^ spikes that were not blocked by TTX. The mean latency to spiking after application of PTIO was 2.4±1.3 (SE) min.

The depolarization of B31/B32 *in situ*, and the direct depolarization of B31/B32 in culture by PTIO, might have been caused by possible effects of PTIO that are not related to its inhibition of NO. To eliminate this possibility, we examined the effects of blockers of nitrergic transmission that operate via a different mechanism. We examined the effect on isolated B31/B32 neurons of L-NAME, a competitive inhibitor of NOS, which produces NO from L-arginine [1]. In 11 of 11 preparations, application of L-NAME depolarized B31/B32 and caused firing (Fig. 3B). The latency from application of L-NAME to spiking in B31/B32 was 7.65±2.07 (SE) min, somewhat longer than for PTIO, as might be expected for a substance that does not directly act on the already-released NO. By contrast, in 4 of 5 preparations, D-NAME, the enantiomer of L-NAME that does not affect NOS, had no effect on isolated B31/B32 neurons (Fig. 3C). Application of L-NNA, another competitive inhibitor of NOS, also depolarized and caused B31/B32 firing (Fig. 3D) in 4 of 4 preparations, with a mean latency to spiking of 7.0±1.9 (SE) min, comparable to that caused by L-NAME. The three blockers of nitrergic transmission depolarized B31/B32 by a mean of 24.9±8.9 (SD) mV (Fig. 3F) before the neuron began to fire. In these experiments the depolarization and firing were not terminated by the endogenous K^+^ currents, since firing in B31/B32 neurons autaptically excites the neurons [24], overcoming the effects of the K^+^ currents. The autaptic excitation is blocked in the recording in TTX shown in Fig. 2B. A summary of the effects of the NO blockers, and of the effects of ASW and D-NAME controls, is shown in Fig. 3F.

In experiments on cultured B31/B32 which fire after treatment with NO blockers, the firing was generally terminated by washing out the blocker a few seconds after the start of the firing. However, in some experiments the depolarization and firing was allowed to continue for up to 2 min. There was no repolarization of B31/B32, since in situ repolarization is dependent on synaptic input that inhibits B31/B32 [38], [39]. Neurons producing this inhibition were not co-cultured with B31/B32.

Since NO is synthesized by NOS from L-arginine, increases in L-arginine concentration should increase the activity of NOS, and thereby overcome the excitatory effect of L-NAME. In cultured, isolated B31/B32 neurons that had been depolarized by treatment with L-NAME, in 5 of 5 preparations subsequent treatment with L-arginine repolarized the B31/B32 neurons (Fig. 3E). In these experiments, the L-arginine was applied before the L-NAME had completely depolarized B31/B32 and had initiated firing.

The ability of nitrergic blockers to depolarize B31/B32 neurons that were cultured in isolation was surprising, since no other cells were present that could be releasing NO. These experiments strongly suggest that B31/B32 neurons are themselves nitrergic, and produce NO at rest, in the absence of firing and Ca^+2^ entry into the cell. Production of NO by B31/B32 at rest causes self-inhibition of B31/B32. L-NAME and L-NNA blocked the tonic self-inhibitory NO production, and thereby excited the neuron. PTIO reduced the NO produced tonically, and thereby also excited the neuron.

### Block of guanylyl cyclase depolarizes B31/B32

**Figure 4:**
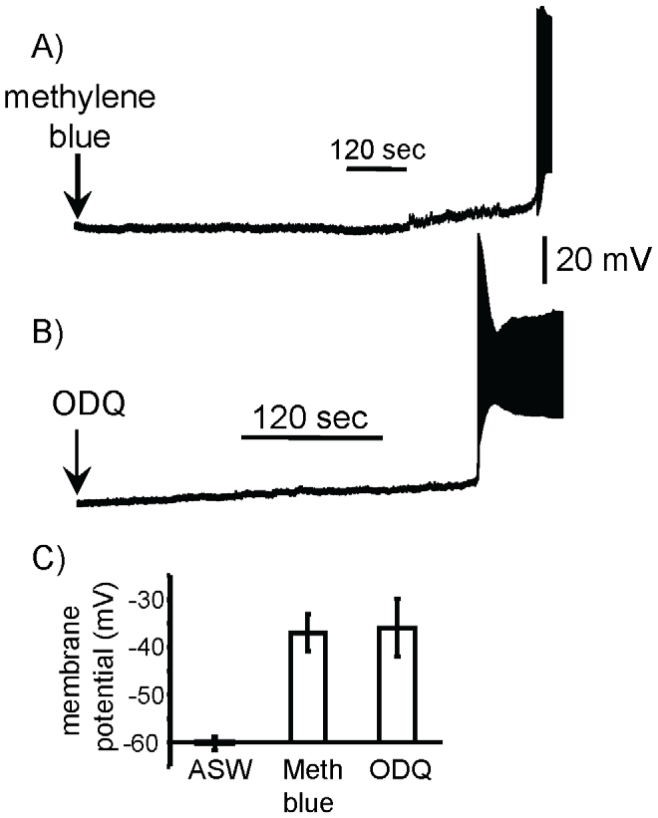
Blockers of guanylyl cyclase depolarize isolated B31/B32 neurons. In the recordings shown, pharmacological agents were applied at the arrow, with the resting potential preceding treatment at −60 mV. **A**) The guanylyl cyclase blocker methylene blue depolarized B31/B32 in 5 of 5 preparations. Mean latency to spiking: 19.6±1.4 (SE) min. Mean amplitude of depolarization: 23.0±3.3 (SE) mV. **B**) The guanylyl cyclase blocker ODQ depolarized B31/B32 in 5 of 5 preparations. Mean amplitude of depolarization: 24.0±6.06 (SE); mean latency to spiking: 3.2±2.94 (SE) min. **C**) Summary of the effects of ASW (N = 3), and of the 2 blockers of guanylyl cyclase on the membrane potential of B31/B32. For the substances causing firing of B31/B32, the potential just before the first spike is shown. Means and standard errors are shown.

Effects of NO are often mediated via the activation of guanylyl cyclase and the synthesis of cyclic GMP (cGMP), which acts as a second messenger in effecting cellular changes. To strengthen the finding that B31/B32 displays self-inhibitory NO production at rest, we applied inhibitors of guanylyl cyclase to isolated, cultured B31/B32 neurons. The effects of both methylene blue [4], [8], [40], [41] and 1H-[1,2,4]Oxadiazolo[4,3-a]quinoxalin-1-one (ODQ) were tested. Application of either methylene blue (Fig. 4A) or of ODQ (Fig. 4B) caused depolarization and firing of cultured, isolated B31/B32 neurons similar to that induced by blocking nitrergic transmission. This finding is consistent with a continuous synthesis of cGMP that contributes to the resting potential of B31/B32, since block of guanylyl cyclase in the absence of additional stimuli depolarized the cell. The continuous synthesis of cGMP is consistent with continuous production of NO.

### Currents underlying nitrergic self-inhibition

**Figure 5:**
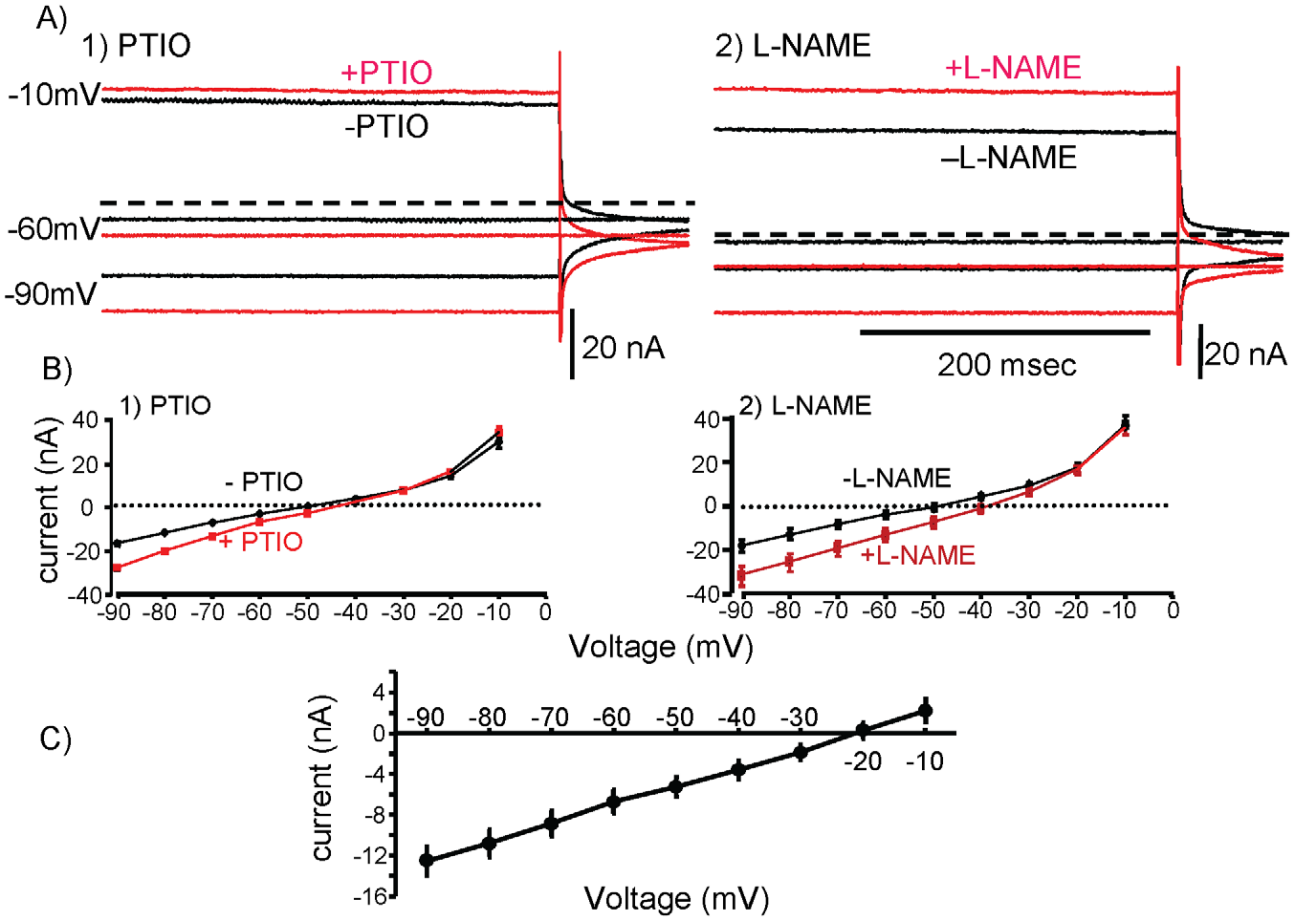
Block of NO opens an inward current. **A**) Effect of PTIO and of L-NAME on currents recorded during the last few hundred milliseconds of a voltage clamp experiment performed in TTX. Only currents recorded in response to voltage steps to −90, −60 and −10 mV are shown. Note that both PTIO and L-NAME induce inward currents at −90 and −60 mV, with the currents at −90 mV larger than those at −60 mV. Also note the reversal of the currents at −10 mV. **B**) Mean and standard errors (hidden by the points) of current amplitudes recorded during the last 500 msec of voltage pulses with and without PTIO or L-NAME (N = 5 for each group). **C**) The difference in current between values recorded with and without PTIO or L-NAME at the various voltage steps. The data were combined from experiments using the two blockers. Means and standard errors are shown.

What type of ion channels are present in B31/B32 that respond to NO, and that depolarize the neuron when NO is blocked? To characterize the channels underlying the response of B31/B32 to blocking NO, B31/B32 neurons were voltage-clamped in the presence and absence of either PTIO or L-NAME. As described previously [28], B31/B32 neurons were impaled with two electrodes, one for passing current, the other for measuring the voltage. The experiment was performed on B31/B32 neurons *in situ* in response to 3 sec command pulses ranging from −90 to −10 mV. Spikes were blocked with tetrodotoxin (TTX). The effects of PTIO and L-NAME were quantified over the last 100 msec of the pulse (Fig. 5A), by subtracting currents in the presence of the NO blockers from those in ASW (Fig. 5C). A previous study [28] showed that the B31/B32 somata contain two active outward currents that both have an activation threshold of approximately −40 mV, and which show time-dependent inactivation. Neither current could contribute to the steady-state inhibition of B31/B32, since the currents are not active at rest. Both currents are largely inactivated by end of a 3 sec voltage pulse, and they do not contribute to measurements of the effects of PTIO and L-NAME at the end of the 3 sec voltage pulses.

Similar results were obtained with both PTIO and L-NAME, which block nitrergic transmission via different modes of action. Treatment with either PTIO or L-NAME produced a net inward current (Fig. 5A). From −90 mV to −10 mV the current was not voltage-dependent (Figs. 5C). PTIO and L-NAME both caused a net increase in conductance of approximately 5 mS. Combined data from recordings in PTIO and L-NAME (Fig. 5C) showed that blocking nitrergic transmission unmasked a leak current with a −20 mV reversal potential.

### Nitrergic self-inhibition is not an artifact of culturing B31/B32

**Figure 6:**
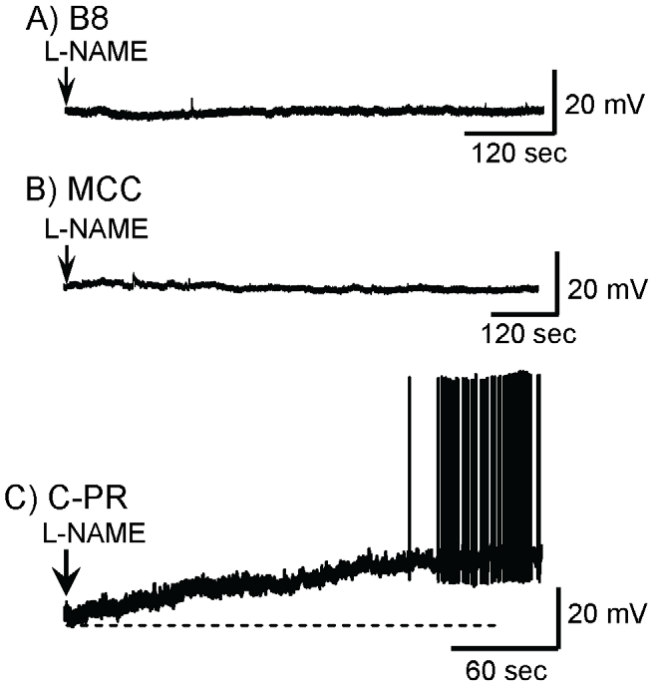
Effects of NO blockers and donors on other neurons. **A**) L-NAME had no effect on neuron B8 in 6 of 6 preparations. L-NAME also had no effect on B4 in 5 of 5 preparations (not shown). **B**) Treatment of an isolated MCC neuron in culture with L-NAME had no effect in 7 of 7 preparations. **C**) In an isolated, cultured C-PR neuron application of L-NAME caused a depolarization in 5 of 5 preparations. Mean amplitude of depolarization: 13.3±1.07 (SE) mV; Mean latency to spiking: 7.11±1.8 (SE) min. The dashed line marks the −60 mV resting potential.

Although nitrergic background inhibition was observed in intact buccal ganglia (see Figs. 1, 2), it was important to be certain that our central finding, that background self-inhibition is also found in isolated, cultured B31/B32 neurons (Fig. 3), did not arise as a result of changes in the properties of the neuron when it is cultured in isolation. To rule out this possibility, we examined whether similar nitrergic self-inhibition is also seen in other isolated, cultured neurons that control aspects of feeding. Neither L-NAME (Fig. 6A) nor PTIO (not shown) had an effect on buccal ganglia neuron B8. L-NAME also had no effect on buccal ganglia neuron B4 (not shown), or on cerebral ganglion neuron MCC (Fig. 6B). These data show that not all isolated, cultured *Aplysia* buccal or cerebral ganglia neurons respond to L-NAME.

### Cerebral ganglion arousal neuron C-PR also displays nitrergic self-inhibition

**Figure 7:**
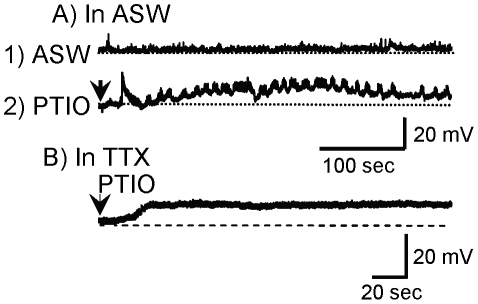
Block of NO depolarizes C-PR in situ. **A**) Application of the NO scavenger PTIO in an isolated cerebral ganglion preparation depolarized C-PR and caused an increase in EPSPs. **B**) In the presence of TTX, PTIO still depolarizes C-PR (N = 5 cells in 3 preparation), indicating that part of the effect is direct.

We also tested the effects of L-NAME on neuron C-PR of the cerebral ganglion. C-PR polysynaptically excites the MCC and additional neurons active during head-waving and biting [40], [41], and C-PR has been characterized as a command neuron for a behavioral state, food arousal [30]. Treatment with L-NAME caused depolarization and firing of isolated C-PR neurons (Fig. 6C). Since no other neuron was present, this experiment demonstrates that C-PR also contains an isoform of NOS that is active and produces NO at rest, in the absence of firing. As in B31/B32, L-NAME blocked the self-inhibition caused by NO production from C-PR onto itself at rest, and thereby excited the neuron. Depolarization of C-PR was also found when recording from the neuron in situ when PTIO was applied to the cerebral ganglion (Fig. 7), indicating that the excitatory effect on C-PR of blocking NO is not an artifact of culturing. In these experiments, PTIO increased excitatory synaptic outputs onto the cell (Fig. 7A), indicating that part of the tonic nitrergic inhibition of C-PR is indirect, via inhibition of neurons that synaptically excite C-PR. However, some of the effect of PTIO *in situ* is also direct, since the depolarization is also seen after treatment with TTX (Fig. 7B), which blocked firing in presynaptic neurons.

## Discussion

We have presented data in support of a novel mechanism by which NO regulates the nervous system. In the absence of action potentials, NO is produced by at least two key neurons that control behavior, C-PR and B31/B32 (Figs. 3, 6). In these neurons the tonic presence of NO causes self inhibition and a stabilization of the resting potential.

C-PR has been characterized as a command neuron eliciting a central motive state, food arousal [30]. C-PR is excited by food on the lips [30], and its firing induces the head lifting component of head-waving [42], a behavior by which *Aplysia* localize the position of food, before locomoting toward the food [2]. C-PR also polysynaptically excites the MCC, a neuron that effects aspects of food arousal [29], as well as exciting command-like interneurons [30], [43] that recruit the CPG organizing repetitive bites [43], [44]. Since inhibiting nitrergic transmission depolarized and caused firing of C-PR, one would predict that in intact, behaving animals inhibitors of nitrergic transmission will induce food-finding behaviors such as head-waving, as well as consummatory behaviors, such as biting. These predictions have been confirmed [25].

B31/B32 are key components of the buccal ganglia CPG controlling consummatory feeding behaviors [22], [26]–[28]. B31/B32 are active during the protraction phase of consummatory behaviors [27], and depolarizing or hyperpolarizing the cells respectively increases or decreases the rate of fictive consummatory responses [26]. The properties of the cells indicate that the decision of whether or not to initiate a consummatory behavior is made by these cells [28]. The initiation of buccal motor programs (Figs. 1, 2) after treatment with blockers of nitrergic transmission is consistent with the effects of blocking the stabilization of the B31/B32 resting potential. Data presented elsewhere [25] confirm that treating intact animals with blockers of nitrergic transmission also initiates consummatory feeding behaviors.

The findings that C-PR and B31/B32 are nitrergic is consistent with previous morphological studies that characterized nitrergic neurons and processes in *Aplysia* [13], [14], [45]. These studies showed heavy staining of nitrergic processes in the area of B31/B32 and C-PR.

Background nitrergic inhibition is not restricted to B31/B32 and C-PR. NO scavengers applied to the cerebral ganglia induced increases synaptic excitation onto C-PR (Fig. 7), indicating that neurons presynaptic to C-PR are depolarized and fire in response to blocking nitrergic inhibition. In addition, some buccal ganglia motor neurons also contain NO [46], suggesting that NO is tonically produced in these neurons.

### Specificity of pharmacological effects

Our findings are largely based on the pharmacological effects of nitric oxide blockers, such as L-NAME, L-NNA and PTIO. These could have effects not related to their inhibition of nitrergic transmission. However, a number of points strongly support the likelihood that the effects seen result from changes in nitrergic transmission. First, both competitive inhibitors of NOS and an NO scavenger were used in different experiments, and sometimes in the same experiment (Figs. 3, 5). Although these agents have different modes of inhibiting nitrergic transmission, their effects were consistent across experiments, or produced similar effects in the same experiment. Second, L-arginine, the precursor of NO, had an inhibitory effect opposite to the excitatory effects a NOS inhibitor (Fig. 3E). Third, blocking guanylyl cyclase, which in many systems is activated by NO [1], caused depolarization of B31/B32 similar to that caused by blocking NO. Fourth, since pharmacological agents blocking NO had similar effects on both B31/B32 and on C-PR (Figs. 1, 2, 3, 6, 7), it is unlikely that an effect is an anomaly arising from the unusual properties of a specific neuron, or of an artifactual effect of an agent at a specific site. Fifth, blockers and donors of NO had effects on behavior in intact animals that were consistent with the effects documented above on neural activity [25].

### Cellular mechanism of nitrergic self-inhibition

Neuronal NO production depends on spike-dependent Ca^+2^ entry, which activates NOS [1]. However, NO is produced without spikes by B31/B32 and C-PR. In situ, blocking Na^+^-dependent spiking with TTX did not affect the background inhibitory effect of NO, since an NO scavenger was still effective in depolarizing B31/B32 and C-PR. In addition, an NO scavenger and an NOS blocker depolarized B31/B32 and C-PR when they were cultured in isolation and were silent, at rest potential. These neurons may have a Ca^2+^-insensitive NOS isoform (*e.g.*, [14]), there could be Ca^+2^ leak at rest, or resting Ca^2+^ concentrations may be sufficient to activate NOS, as in other tissues [47], [48].

Experiments blocking background NO effects in B31/B32 de-suppressed a constitutive inward leak current which depolarizes the cells when NO is blocked (Fig. 5). A depolarizing leak current has been cloned in mice [49], and such a current is present in *Lymnaea* [50]. Our results suggest that such a current may be present in *Aplysia*. The finding that blocking guanylyl cyclase also depolarizes B31/B32 suggested that NO acts via guanylyl cyclase, as it does in many other systems. If so, the inward current would be inhibited by NO via the activation of guanylyl cyclase and the production of cGMP.

Although a background self-inhibitory nitrergic inhibition in the absence of spikes is to date a unique phenomenon, it has features in common with processes occurring in vertebrate retinal receptor cells. In these cells, the tonic presence of cGMP opens a channel which depolarizes the cell [51], rather than closing such a channel and thereby preventing depolarization, as in B31/B32. Thus, the control of the membrane potential in B31/B32 and in the retina is via cGMP, but cGMP has opposite effects in the two tissues. Nitrergic self-inhibition also has features in common with nitrergic excitation of the MCC in *Aplysia* [7], in which NO via cGMP closes an outward leak channel, thereby depolarizing the MCC, rather than closing an inward leak channel and thereby polarizing B31/B32.

### L-arginine is likely to act by regulating background NO

The existence of background inhibitory NO production in neurons controlling aspects of feeding raises a question of function. Why actively inhibit feeding-related neural activity via the constant production of NO at rest, in the absence of stimuli that elicit the behavior? What is there to actively inhibit if there are no stimuli eliciting feeding behavior? A trivial potential answer is that nitrergic inhibition of C-PR and B31/B32 is merely an unusual mechanism for generating the resting potential, a general property of all cells. However, the expression of this unusual cellular mechanism in key neurons organizing different aspects of feeding behavior, localized in different ganglia, suggests that it may function to allow a circulating molecule to regulate NO production, and thereby regulate different aspects of feeding behavior.

Another study [25] is consistent with this suggestion. This study showed that physiological increases in the hemolymph concentration of the amino acid L-arginine can inhibit feeding. The regulation of feeding by L-arginine provides a plausible function for the presence of nitrergic self-inhibition in neurons C-PR and B31/B32. L-arginine is the precursor from which NO is synthesized. L-arginine is generally present in concentrations that are sufficient for the production of NO when NOS is activated. In most nitrergic neurons, NOS will be transiently active in response to Ca^+2^ entry resulting from spiking. Because NOS is transiently active, a tonic increase in L-arginine concentration could transiently lead to a brief increase in NO concentration, which extends the active radius of NO release, as well as more strongly affecting cells already within the active radius. However, in C-PR and in B31/B32, in which NOS is likely to be continuously active, the increased L-arginine will cause a continuous increase in NO synthesis, potentially allowing even a small increase in L-arginine to have a continuous effect. In addition, since the neurons producing NO are themselves sensitive to NO, the maximal effect of the increased NO synthesis will be at the site of the NO synthesis. Thus, building neurons with continuously active, self-affecting NO production is a highly effective way to regulate their activity by changes in L-arginine.

Although it is not mediated by NO, background synaptic inhibition is also a feature of the CPG controlling *Lymnaea* feeding [52], indicating that inhibitory modulation of a feeding CPG may be a general control feature. In this system, an inhibitory neuron that is part of the CPG tonically fires at rest, and thereby inhibits the CPG. Just as the tonic NO is release is overcome by food-related stimuli in *Aplysia*, the tonic firing in *Lymnaea* is suppressed when feeding is initiated.

### Guanylyl cyclase as a modulator of feeding strategies

Inhibitors of guanylyl cyclase depolarize isolated B31/B32 neurons (Fig. 4), suggesting that nitrergic self-inhibition, and the regulation of feeding via L-arginine [25], is via the cGMP second messenger pathway. cGMP is a modulator of feeding in other systems [53]. Variations in genes governing PKG, the kinase responding to cGMP, affects feeding strategies in *Drosophila* [54], and in other insects [55]–[57], as well as in *C. elegans* [58] and possibly in vertebrates [53]. Control of feeding by cGMP in different phyla suggests that such control may have been present in early multi-cellular animals, and is preserved with variations in descendents in different phyla.

### Elicited NO release also has predominantly inhibitory effects on feeding

In addition to acting as a background factor inhibiting key neurons driving feeding, NO is released in *Aplysia* in response to a number of stimulus conditions. Conditions causing an increase in NO also generally inhibit feeding, suggesting that background and elicited effects of NO are consistent. For example, NO is a transmitter of the L29 neurons that facilitate withdrawal reflexes [59]. NO is also elicited by stimuli causing tissue damage [41]. Noxious stimuli initiating withdrawal or causing tissue damage also inhibit feeding [60]. Treating *Aplysia* with an NO donor induces egg-laying [61], which also inhibits feeding [62].

NO is released by neuron C2 [6], which responds to food-related stimuli, particularly to stimuli causing attempts to consume a tough food [63]. An adaptive response to tough food is initially to try hard to consume it, but if such attempts fail, animals then reject the food and eventually to stop feeding. NO release may partially underlie the early facilitation of feeding, as animals try to consume the tough food, as well as the later inhibition of feeding. The early facilitation may be via the connection from cerebral ganglion neuron C2 to the MCC. C2 [64] is a sensory neuron that fires in response to attempts to swallow [63]. It excites the MCC, which effects aspects of food arousal [29], by releasing both NO and histamine [6], [64], [65]. NO also functions in biasing feeding responses toward rejection. Thus, treatment with L-NAME causes rejection responses to become more irregular [8]. In addition, when animals are fed a tough inedible food, treatment with L-NAME reduces rejections and causes increased attempts to swallow the food [9]. In learning that food is inedible, a learning paradigm affecting *Aplysia* feeding, NO substitutes for efforts to swallow [8], [10], a necessary component for memory formation [9], suggesting that NO functions in signaling such attempts. Learning is expressed as increased rejection responses and an eventual cessation of attempts to feed [66], which are consistent with the inhibitory effects of NO on feeding. Inhibition of feeding caused by NO released as a result of efforts to swallow could act at the same sites responding to the background tonic inhibitory NO production. Efforts to swallow will release much more NO, and will produce a much stronger feeding inhibition, than the feeding inhibition caused by background NO production.

In *Lymanea* blocking NO blocks responses to food, presumably because taste afferents are nitrergic [4]. *Lymnaea* feeding is characterized by rasps [21], rather than by separate ingestion and rejection feeding responses. Rasps in *Lymnaea* may be excited by factors such as NO that facilitate rejection in *Aplysia*. In addition, in *Lymnaea* increased NO levels inhibit fictive feeding [11], [12]. Thus, NO may have mixed effects on *Lymnaea* feeding.

### Background nitrergic modulation in other systems

Background nitrergic modulation of neurons is also found in other systems. However, two aspects of nitrergic inhibition of *Aplysia* feeding are to date unique: 1) NO is produced in neurons in the absence of spiking; 2) NO causes self-inhibition by blocking an inward leak current.

Inhibiting NO destabilizes the crab stomatogastric ganglion [20], which contains two networks. After blocking either NO or guanylyl cyclase the networks combine into a single circuit. Many neurons in the ganglion are nitrergic, suggesting that NO released from the CPG modulates it. In the crustacean neurogenic heart, NO released from the heart exerts inhibitory control on the cardiac ganglion CPG [17]. NO also inhibits the locomotor pattern generator in tadpoles [18], [19], [32], where NO increases the effects of inhibitory interneurons, and depolarizes motor neurons by closing a K^+^ channel, which increases their input resistance.

Background NO release affecting the nervous system without neural activity is found in the hippocampus, where both elicited and background NO release facilitate long-term potentiation (LTP) [16], [66]. In this system, background and elicited NO are released from different tissues by different mechanisms. Background NO is released from endothelia lining blood vessels that are in proximity to neural targets, whereas elicited NO release occurs as a result of action potentials in neurons [48]. Release of NO from capillaries onto neurons also occurs in the optic nerves of mammals [67].

## Materials and Methods

### Animals

*Aplysia californica* (5–200 g) were purchased from Marinus Scientific (Garden Grove, CA), Santa Barbara Marine Bio (Santa Barbara, CA) and from the NIH/University of Miami National Resource for *Aplysia*. Animals were maintained on a 12 hours light-dark cycle in 900 liter tanks of aerated, filtered Mediterranean seawater at 18°C. They were fed every 3–4 days with *Ulva lactuca* gathered fresh from the Mediterranean Sea and then kept frozen until needed.

### Pharmacology

Concentrations used were as follows: for the nitric oxide synthase (NOS) inhibitor Nω-nitro-L-arginine methyl ester (L-NAME), 0.37 mM; for a second competitive NOS inhibitor L-N^G^-nitroarginine (L-NNA), 1 mM; for the enantiomer of L-NAME, Nω-nitro-D-arginine methyl ester (D-NAME), 0.37 mM; for the NO scavenger 2-phenyl-4,4,5,5-tetramethyl-imidazdine-1-oxy-3-oxide (PTIO), 1 mM; for tetrodotoxin (TTX), 60 µM; for the guanylyl cyclase blocker methylene blue, 100 µM; for a second guanylyl cyclase blocker 1H-[1,2,4]Oxadiazolo[4,3-a]quinoxalin-1-one (ODQ), 20 mM (ODQ was dissolved in DMSO before placing it ASW). The substances were added to solutions of artificial seawater (ASW) whose composition was (in mM): NaCl, 460; KCl, 10; CaCl_2_, 11; MgCl_2_, 55; and NaHCO_3_, 5 mM; pH = 7.64. The concentrations used for L-NAME, D-NAME, L-NNA, and PTIO were chosen because previous studies [8], [14], [41], [61] showed the efficacy of these concentrations. Chemicals were purchased from Sigma, Israel.

In isolated cultured neurons, nitrergic transmission was blocked by either an NO scavenger or by competitive NOS inhibitors. In acutely dissected preparations, only an NO scavenger was consistently used. Acute preparations require extensive dissection causing tissue damage, which releases NO [41]. In this condition, preliminary experiments showed that a scavenger is more effective than is a competitive inhibitor of NOS.

### Acute extracellular recording

Animals were anesthetized with isotonic MgCl_2_ (25–50% of the body weight) prior to dissection. The buccal ganglia were removed and placed in a chamber containing artificial seawater (ASW). Fictive feeding was recorded via suction electrodes that were placed on the cut end of the radula nerve (RN) and buccal nerve 2 (BN2). RN and BN2 were chosen because recordings from these nerves are useful monitors of ingestion versus egestion-like patterns of fictive feeding [31].

### *In situ* current and voltage clamping

After the animals were anesthetized, either the cerebral ganglion or the buccal ganglia were removed and placed in a chamber containing artificial seawater (ASW). The connective tissue sheath overlying neurons was surgically removed. Recordings were at room temperature with 1M KCl electrodes (10–20 MΩ on neurons *in situ*; 40–70 MΩ on neurons in cell culture), via an Axoclamp 2 voltage clamp/amplifier (Axon Instruments) used in either current clamp mode or in two-electrode voltage clamp mode, as appropriate to the design of the experiment.

### Cell culture

Cerebral or buccal ganglia were dissected, and then bathed in protease (Sigma, Israel) for 2 hours. Ganglia were desheathed, the cells of interest were identified via intracellular recording, and then removed from the ganglion and cultured for 3–4 days at 18° in hemolymph and L15 (Sigma) with salts added to adjust the salinity to that of seawater, as described previously [68], [69]. Recordings were in 50% ASW- 50% L-15.
